# Fractures of Lower End Humerus Involving the Elbow

**Published:** 1914-06

**Authors:** W. E. Person

**Affiliations:** Atlanta, Ga.


### FRACTURES OF LOWER END HUMERUS INVOLVING THE ELBOW.

By W. E. Person, Atlanta, Ga.

While this is a subject familiar to all of you, the fact that it frequently causes much trouble and dissatisfaction, and the hope that in the discussion many good points will be brought out, is the reason for this paper:

In every case of injury in the neighborhood of the elbow accompanied by pain, swelling and more or less loss of function, accurate diagnosis is essential to secure a good result. According to Scudder, the following must be borne in mind:

Fracture shaft just above condyles.
Fracture internal epicondyle.
Fracture internal condyle.
Fracture external condyle.
T Fracture.
Dislocation backward elbow.
Fracture olecranon.
Fracture Coronoid process.
Fracture radial neck.
Subluxation radius.

When convenient, the X-ray gives positive information, but in the majority of cases an anesthetic must be used for diagnosis. The normal limb should always be compared with the injured one. The examination should be systematic and thorough. Measurements from the tip of the acromion to the external condyle and from the external condyle to the inner give valuable information as to shortening of arm and widening of elbow. Three bony points of great importance are internal and external condyles, and the olecranon. In a normal elbow an imaginary line passed through the condyles just below the tip of the olecranon when the forearm is extended. When the forearm is flexed the three points are in the same posterior plane. Lateral elbow motion does not exist in a normal joint. It is only present in fracture of the humerus when near the joint.

Supra condylar and internal epicondylar fractures will not be considered in this paper.

Fracture of the internal condyle causes more swelling at this point, the carrying angle is destroyed, there is lateral motion in adduction, and owing to the action of the flexor group of muscles of the forearm pulls the condyle downward and forward disturbing the relation of the three bony points. The fragment moves freely upon the seat of the bone and crepitus is easily obtained.

Fracture of the external condyle has the swelling most marked there. The condyle is usually pulled upward causing the relation of the bony points to be changed. Abnormal motion and crepitus locate the site of the fracture.

T-fracture gives lateral motion in adduction and abduction. The transverse measurement is increased. The bony landmarks are not in normal relation to each other. The fragments move upon the shaft and upon each other. Crepitus is easily felt.

The epiphyseal fracture is found in children. The diagnosis is made from the age, abnormal motility low down on the shaft, more in an antero posterior direction than laterally, and crepitus which has been described by Poland as muffled.

The prognosis should be somewhat guarded as there is always possibility of partial or complete ankylosis and an ugly deformity is often seen.

Da Costa says, "Ankylosis may be due to the prolonged immobilization, the muscles becoming contracted and fibrous, the fascia and ligaments about the joint shortening and the capsule shrinking and thickening, some of the cartilages becoming fibrous and the joint partly obliterated. It may result from hemorrhage into the joint and tendon sheaths with subsequent formation of fibrous tissue. The organization of inflammatory tissue in the joint, tendon and muscle sheaths. Excessive callous might limit or block motion. This excess is due either to an imperfect reduction or too much movement of the fragments."

One thing that is comforting is the fact that a deformity does not bar a good functional result. The carrying angle is not essential for good service.

Treatment:—An anesthetic should always be given. With the exception of a few cases where gangrene is threatened, I do not think it advisable to use a temporary dressing. By a prompt reduction and application of suitable splint the patient is saved pain, time and his chance of a perfect result is enhanced. Swelling subsides more rapidly when the limb is put up in plaster of Paris than when allowed to lie in a trough of a pillow. As a splint, plaster of Paris has given the writer better service than any other material. I have heard it criticized, when the criticism should have been placed elsewhere. Before applying it the skin and superficial bony points should be well protected by a soft bandage of cotton. The plaster roll should be put on to hold the parts in position, not to make pressure.

In epiphiseal fracture by extension of the forearm and counter extension of the arm and manipulation proper alignment is obtained. The forearm is supinated and put up at a right angle.

The fractures of the condyles and of the T variety are reduced by the forearm being in extension, by grasping the condyles between the thumb and fingers of one hand and the forearm by the other, traction is made of the fragments, while an assistant exerts a counter pull upon the arm. Next the supinated forearm is flexed to an acute angle and by manipulation the pieces are brought into a satisfactory position. The degree of flexion varies in inverse proportion to the amount of swelling.

This position I find holds the fragments in place better than any other and has the advantage of having a useful member in case of ankylosis. Furthermore, it is easier and less painful to acquire extension than flexion.

The plaster of Paris splint should extend from the base of the fingers up the shoulder, making a cap for the joint and supporting the limb in a wide sling. In many cases it is better to carry the plaster over the chest, making a spica bandage. The fingers should be left so that free movement can be made, in fact, it should be encouraged.

For the first 24 or 48 hours we should keep in close touch with the patient and any constriction noted. The signs of which are increased pain, swelling, cyanosis and coldness of the fingers. Free movement of them is also diminished. If these appear the splint should be cut through the skin and sprung open.

Early resumption of the daily routine and performance of what duties can be done should be advised as this means a more rapid convalescence.

After reduction an X-ray should be made as a check on our work, and if the position is not satisfactory the bones should be reset.

The length of time the cast stays on depends on the amount of swelling and how loose it becomes. In an ordinary case it should be taken off in about two weeks, passive motion is cautiously used, and another one applied. The last one is allowed to stay two or three weeks and then if union is good a sling is substituted.

Passive motion is now to be commenced and should always be made by the doctor. Fexion, extension, supination and pronation should be persistently used. This should be done at least every other day. In treating children the parents should be told the necessity of this even if it causes pain and resistance. Every means should be tried to induce them to use the limb in play or work. Lifting weights, etc., do great good.

Through all these procedures the sound limb should be taken as a model and effort directed to make the injured one equal in symmetry and function.

Candler Bldg., Atlanta, Ga.

